# Determination of 35 Free Amino Acids in Tea Using Ultra-Performance Liquid Chromatography Coupled With Quadrupole Time-of-Flight Mass Spectrometry

**DOI:** 10.3389/fnut.2021.767801

**Published:** 2021-12-01

**Authors:** Jian Li, Junmei Ma, Qiang Li, Sufang Fan, Lixin Fan, Hongyu Ma, Yan Zhang, Lei Zheng

**Affiliations:** ^1^School of Food and Biological Engineering, Hefei University of Technology, Hefei, China; ^2^College of Applied Arts and Science, Beijing Union University, Beijing, China; ^3^Hebei Food Safety Key Laboratory, Hebei Food Inspection and Research Institute, Shijiazhuang, China; ^4^Hebei Key Laboratory of Forensic Medicine, College of Forensic Medicine, Hebei Medical University, Shijiazhuang, China

**Keywords:** ultra-performance liquid chromatography, quadrupole-time of flight mass spectrometry, tea, free amino acids, detection

## Abstract

The purpose of this research was to develop a simple, sensitive, and accurate method for simultaneous determination of 35 free amino acids using ultra-performance liquid chromatography coupled with quadrupole time-of-flight mass spectrometry (UPLC-Q-TOF/MS). Tea samples were extracted with boiling water bath, and then separated by XBridge BEH Amide column by gradient elution. The exact mass and MS/MS spectra of the target compound was detected under the TOF–MS and Information dependent acquisition (IDA)–MS/MS mode. The results demonstrated good linearity (*R*^2^ > 0.9980) in the range of 0.5–1,000 ng/mL. The limits of detection (LODs) were 0.13–25.00 mg/kg and the limits of quantitation (LOQs) were 0.25–50.00 mg/kg. The recovery rate ranged from 70.1 to 105.1% with relative standard deviations (RSDs) <11% (*n* = 6). This research provides a targeted strategy for developing an analysis method for amino acids in tea.

## Introduction

Tea, which originated in China, is one of the most popular and widely consumed beverage in the world because of its refreshing taste, attractive aroma, and potential health benefits (1). The production and processing of tea have a significant impact on the economic and social development in China due to the huge economic benefits associated with it. In recent years, there has been extensive research on the valorization and health benefits of medicinal plants (in general) and teas (in particular), but systematic and in-depth research on the chemical components of flavor in tea is still weak in China (2). Amino acids and other molecules such as carotenoids, fatty acids, glycosides, and sugars contribute to the aroma in teas (3, 4). Free amino acids play an important role in the chemical composition of tea (5), which is one of the important evaluation factors of tea quality (6). Amino acids are the basic units of proteins, which are also the important components of active peptidases and other bioactive molecules (7–9). The composition and content of amino acids and their degradation products and transformation products also directly affect the quality of tea. Amino acids participate in the formation of aroma in tea processing, that is because amino acids converted into volatile aldehydes or other products during tea processing, forming tea aroma. In addition, some amino acids themselves have a certain fragrance and are among the most important ingredients that contribute to the quality of tea. For example, theanine, which is a unique amino acid in tea, can relieve the bitterness and astringency of the tea itself due to its fresh smell and caramel aroma, and is the main ingredient that produces sweetness in tea (10). Alanine and glutamic acid have floral aroma characteristics. Serine and tyrosine have wine aroma characteristics (11). The free amino acids in tea participate in the formation of tea color, aroma, and taste through a variety of ways (12). The content and combination of ingredients have a direct impact on the taste of tea soup and the quality of tea (13), which requires accurate and rapid amino acid detection technology.

In recent years, many scholars have devoted themselves to the research on the detection technology of amino acids in tea, and various analytical methods have been proposed including ninhydrin colorimetric method (14–16), amino acid automatic analyzer method (17), high performance liquid chromatography (HPLC) (18–20), and high performance liquid chromatography–triple quadrupole mass spectrometry (HPLC-QQQ-MS) (21–25). Traditionally, the determination of the total amount of free amino acids in tea has been conducted by the ninhydrin colorimetric method, which is stipulated in the national standard (26). However, the limitation of this strategy is that it can only measure the total amount of amino acids and cannot accurately measure the content of individual amino acid components (27). The determination using automated amino acid analyzer is a traditional method for the analysis of free amino acids. The disadvantage of the method is post-column derivatization with long analysis times and low sensitivity (28). At present, high performance liquid chromatography method mostly involves pre-column derivatization technology, in which the measuring procedures are complicated and greatly affected by derivatization reagents (29–32). The accuracy and operability of derivatization need to be further improved. Liquid chromatography–mass spectrometry (LC–MS) is a powerful analytical technique, and depending on the mass analyzer coupled to the separation technique offers other advantages (33). LC-QQQ-MS and LC-QTrap-MS have high specificity but low resolution, and there are problems such as serious matrix interference and little information about compound fragmentation. On the other hand, LC-Orbitrap-MS and LC-Q-TOF/MS approaches have high resolution and accuracy, and have strong anti-interference ability and wide scanning range (34, 35). In this study, UPLC-Q-TOF/MS was used for the first time to detect 35 free amino acids in tea. This method provides technical support for quality control and research and development of tea, and promotes the sustainable development of the industry.

## Materials and Methods

### Reagents and Standards

Acetonitrile was HPLC-MS grade and purchased from Fisher Scientific (Loughborough, UK). Water was HPLC grade and supplied by Watson's Food & Beverage Co., Ltd. (Guangzhou, China). Formic acid was HPLC grade supplied by Sigma–Aldrich (St. Louis, Missouri, USA). Magnesium oxide was purchased from Kemiou Chemical Reagent Co., Ltd. (Tianjin, China). The origin of congou black tea was Hangzhou, Zhejiang Province, China. The origin of Yellow mountain fuzz tip was Huangshan City, Anhui Province, China. The origin of rizhao green tea was Yantai City, Shandong Province, China. The origin of green tea 1 was Suzhou City, Jiangsu Province, China. The origin of green tea 2 was Suzhou City, Jiangsu Province, China. The origin of maojian tea was Hangzhou City, Zhejiang Province, China. The origin of yacca tea was Hangzhou, Zhejiang Province, China. The origin of taiping houkui tea was Huangshan City, Anhui Province, China. The origin of sword-shaped green tea was Shaoxing City, Zhejiang Province, China. All dry tea samples were purchased from local tea shops in Shijiazhuang, China. All samples were stored in dark and dry places at room temperature.

L-Arginine (purity ≥ 99.0%), L-aspartic acid (purity ≥ 99.0%), L-glutamic acid (purity ≥ 99.0%), glycine (purity ≥ 99.5%), L-histidine (purity ≥ 98.0%), L-isoleucine (purity ≥ 99.0%), L-leucine (purity ≥ 99.0%), L-lysine (purity ≥ 98.0%), L-methionine (purity ≥ 99.0%), L-phenylalanine (purity ≥ 99.0%), L-proline (purity ≥ 99.0%), L-serine (purity ≥ 99.0%), L-threonine (purity ≥ 99.0%), L-tyrosine (purity ≥ 99.0%), L-valine (purity ≥ 99.0%), and L-alanine (purity ≥ 99.0%) were supplied from J&K Scientific Ltd. (Shanghai, China). L-Cysteine (purity ≥ 99.4%) was supplied from Dr. Ehrenstorfer (Augsburg, Germany). Theanine (purity ≥ 98.0%), L-tryptophan (purity ≥ 99.0%), L-asparagine (purity ≥ 98.7%), and L-glutamine (purity ≥ 99.5%) were supplied from ANPEL Laboratory Technologies (Shanghai, China). L-cystine (purity ≥ 99.3%), aminoadipic acid (purity ≥ 98.0%), sarcosine (purity ≥ 98.0%), L-pipecolic acid (purity ≥ 98.0%), α-aminobutyric acid (purity ≥ 98.0%), β-aminobutyric acid (purity ≥ 98.0%), citrulline (purity ≥ 98.0%), hydroxylysine (purity ≥ 98.0%), 1-methyl-L-histidine (purity ≥ 98.0%), and 3-methyl-L-histidine (purity ≥ 98.0%) were purchased from Sigma (California, USA). Hydroxyproline (purity ≥ 98.0%), γ-aminobutyric acid (purity ≥ 98.5%), DL-homocysteine (purity ≥ 98.0%), and L-ornithine (purity ≥ 98.0%) were purchased from Chromadex Trading Company (California, USA).

### Instruments

High-speed refrigerated centrifuge (3K13, Sigma, USA), vortex mixer (Vortex Genius 3, IKA, Germany), and magnetic stirrer with heating (RET CV S025, IKA, Germany) were used in the procedure of sample preparation. The separation of compounds were carried out on an UPLC system (LC-30AD, Shimadzu, Japan). Quantitative analysis of 35 free amino acids in tea was conducted on a Q-TOF/MS (TripleTOF^TM^ 5600^+^, Sciex, USA). OS software (Version 1.5.0, Sciex, USA) was used for data processing.

### Methods

#### Sample Preparation

Refer to GB/T 8312-2013 (36) for sample preparation: 0.5 g crushed tea (dry leaves) was weighed into a 500 mL breaker followed by addition of 4.5 g magnesium oxide and 250 mL boiling water. The solution was extracted for 20 min in a boiling water bath. The prepared tea soup was transferred to a 50 mL centrifuge tube and centrifuged for 5 min at 2,128 g at room temperature. The supernatant was filtered through a 0.22 μm filter membrane. When the sample content was too high, it was diluted to the linear range with ultrapure water according to the actual concentration.

#### UPLC Conditions

The chromatographic separation was carried out on a Waters XBridge BEH Amide column (2.1 × 150 mm, 2.5 μm) with a flow rate of 0.4 mL/min. The mobile phases were 0.2% formic acid in water (A) and 0.2% formic acid in acetonitrile (B). The elution gradient was carried out for 20 min as follows: 0–3.00 min, 90% B; 3.0–13.0 min, 90% to 48% B; 13.0–15.0 min, 48% B; 15.0–16.0 min, 48% to 90% B; and 16.0–20.0 min, 90% B. The injection volume was 5.0 μL.

#### Q-TOF/MS Conditions

For the MS analysis, the Triple TOF^TM^5600^+^ equipped with a DuoSpray^TM^ ion source was used. In this study, the electrospray ionization (ESI) source was used for detection and atmospheric pressure chemical ionization (APCI) source was used for calibration. The ionization voltage was set at 5.5 kV and the source temperature at 400°C under positive mode. The curtain gas pressure was 35 psi, the nebulizer gas pressure was 50 psi, and the auxiliary gas pressure was 55 psi. The TOF MS data were collected between *m/z* 20 and *m/z* 500 with a duration time of 20 min, and accumulation time was 0.15 s. The information dependent acquisition (IDA)-MS/MS conditions were as follows: accumulation time was 0.05 s, isotopes within 4 Da were excluded, the declustering potential was 80 V, and the collision energy was 40 ± 20 V. Automatic batch calibration was performed to ensure the accuracy and reproducibility.

#### Database Establishment

Under the instrument conditions given in sections UPLC Conditions and Q-TOF/MS Conditions, 35 amino acid standard solutions were analyzed, and the retention time, accurate mass, isotope distribution, and MS/MS spectrum of each compound were obtained (Table 1). In the Product Ion mode of Triple TOF, when the response value of the target in the retention time window exceeded the threshold, it automatically triggered the acquisition and superimposition of the MS/MS spectrums under different collision energies (20, 40, and 60 eV), thereby establishing a library of MS/MS spectrum of the compound. Import the MS/MS spectrums into the Library module of OS 1.5.0 software to create a mass spectrum database of 35 amino acids.

**Table 1:** Mass spectral parameters for the 35 amino acids.

**Compound name**	**Formula**	**Retention time (min)**	**Adduct/** **charge**	**Theoretical mass (*m/z*)**	**Experimental mass (*m/z*)**	**Mass error (ppm)**	**Isotope ratio difference (%)**
L-Arginine	C_6_H_14_N_4_O_2_	10.190	[M+H]^+^	175.11895	175.11968	4.2	0.4
L-Aspartic acid	C_4_H_7_NO_4_	7.876	[M+H]^+^	134.04478	134.04471	−0.6	0.5
L-Cysteine	C_3_H_7_NO_2_S	6.302	[M+H]^+^	122.02703	122.02692	−0.9	1.5
L-Cystine	C_6_H_12_N_2_O_4_S_2_	10.789	[M+H]^+^	241.03113	241.03148	1.5	2.4
L-Glutamic acid	C_5_H_9_NO_4_	7.557	[M+H]^+^	148.06043	148.06055	0.8	0.8
Glycine	C_2_H_5_NO_2_	6.716	[M+H]^+^	76.03930	76.03962	4.2	1.2
L-Histidine	C_6_H_9_N_3_O_2_	10.282	[M+H]^+^	156.07675	156.07688	0.8	0.3
L-Isoleucine	C_6_H_13_NO_2_	9.908	[M+H]^+^	132.10191	132.10202	0.9	0.1
L-Leucine	C_6_H_13_NO_2_	10.239	[M+H]^+^	132.10191	132.10202	0.9	0.1
L-Lysine	C_6_H_14_N_2_O_2_	10.415	[M+H]^+^	147.11280	147.11278	−0.1	0.8
L-Methionine	C_5_H_11_NO_2_S	4.421	[M+H]^+^	150.05833	150.05836	0.2	1.7
L-Phenylalanine	C_9_H_11_NO_2_	2.420	[M+H]^+^	166.08626	166.08689	3.8	2.4
L-Proline	C_5_H_9_NO_2_	4.620	[M+H]^+^	116.07060	116.07063	0.2	1.6
L-Serine	C_3_H_7_NO_3_	8.149	[M+H]^+^	106.04987	106.04996	0.8	1.4
L-Threonine	C_4_H_9_NO_3_	7.457	[M+H]^+^	120.06552	120.06562	0.8	0.1
L-Tyrosine	C_9_H_11_NO_3_	5.626	[M+H]^+^	182.08117	182.08129	0.6	0.9
L-Valine	C_5_H_11_NO_2_	4.204	[M+H]^+^	118.08626	118.08631	0.4	0.6
L-Alanine	C_3_H_7_NO_2_	6.031	[M+H]^+^	90.05495	90.05502	0.7	1.3
Theanine	C_7_H_14_N_2_O_3_	6.039	[M+H]^+^	175.10772	175.10757	−0.8	0.4
L-Tryptophan	C_11_H_12_N_2_O_2_	4.113	[M+H]^+^	205.09715	205.09657	−2.9	0.6
L-Asparagine	C_4_H_8_N_2_O_3_	8.421	[M+H]^+^	133.06077	133.06076	−0.1	1.7
L-Glutamine	C_5_H_10_N_2_O_3_	8.107	[M+H]^+^	147.07642	147.07622	−1.4	1.1
Aminoadipic acid	C_6_H_11_NO_4_	7.144	[M+H]^+^	162.07608	162.07594	−0.9	1.0
α-Aminobutyric acid	C_4_H_9_NO_2_	4.751	[M+H]^+^	104.07060	104.07050	−1.0	3.3
β-Aminobutyric acid	C_4_H_9_NO_2_	4.095	[M+H]^+^	104.07060	104.07061	0.1	3.9
γ-Aminobutyric acid	C_4_H_9_NO_2_	4.427	[M+H]^+^	104.07060	104.07050	−1.0	3.3
Citrulline	C_6_H_13_N_3_O_3_	8.555	[M+H]^+^	176.10297	176.10302	0.3	0.6
Hydroxyproline	C_5_H_9_NO_3_	6.961	[M+H]^+^	132.06552	132.06550	−0.2	0.5
Hydroxylysine	C_6_H_14_N_2_O_3_	10.953	[M+H]^+^	163.10772	163.10748	−1.5	1.0
1-Methyl-L-histidine	C_7_H_11_N_3_O_2_	8.023	[M+H]^+^	170.09240	170.09261	1.2	3.4
3-Methyl-L-histidine	C_7_H_11_N_3_O_2_	8.293	[M+H]^+^	170.09240	170.09272	1.8	2.5
L-Ornithine	C_5_H_12_N_2_O_2_	10.571	[M+H]^+^	133.09715	133.09692	−1.8	1.0
Sarcosine	C_3_H_7_NO_2_	4.188	[M+H]^+^	90.05495	90.05502	0.7	1.3
DL-Homocysteine	C_4_H_9_NO_2_S	4.821	[M+H]^+^	136.04268	136.04254	−1.0	1.1
L-Pipecolic acid	C_6_H_11_NO_2_	4.344	[M+H]^+^	130.08626	130.08633	0.6	1.0

## Results and Discussion

### Optimization of UPLC-Q-TOF/MS Conditions

Liquid chromatography–mass spectrometry spectra were studied in both positive and negative modes. The results showed that 35 amino acids had higher response in positive mode, and so the positive mode was used for detection in this study. The experiment investigated the response of the 35 amino acids under different declustering potentials (50, 80, 100, 120, and 150 V), and it was found that the amino acid responses were the highest at the declustering potential of 80 V. A lower declustering potential was not conductive to ion transmission, and excessively high declustering potential caused the target compound to fragment within the source. The accurate mass deviations of target compounds were <5.0 × 10^−6^ (Table 1). The MS/MS spectra of the 35 amino acids (Supplementary Figure 1) was used for the final confirmation of the initial screening results.

**Figure 1:**
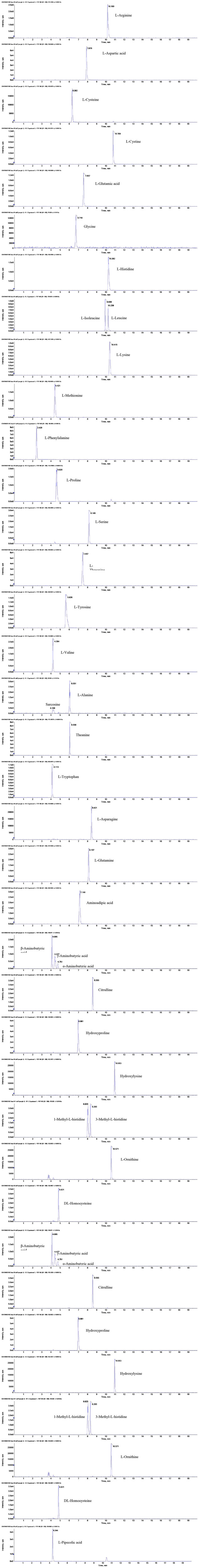
Extracted ion chromatograms of the 35 amino acids precursor ions.

A series of preliminary experiments was carried out using different chromatographic columns including Waters Acquity UPLC HSS T3 (2.1 × 100 mm, 1.8 μm), Waters XBridge BEH C_18_ (2.1 × 100 mm, 2.5 μm), Waters Cortecs UPLC HILIC (2.1 × 100 mm, 1.6 μm), and Waters XBridge BEH Amide (2.1 × 150 mm, 2.5 μm). The results showed (Supplementary Figure 2) that the 35 amino acids were not retained on Waters Acquity UPLC HSS T3 and Waters XBridge BEH C_18_ chromatographic columns. The peak shape obtained on Waters Cortecs UPLC HILIC chromatographic column was poor and wide. The extracted ion chromatograms of the 35 amino acids precursor ions are illustrated in Figure 1. The results showed that the peak shape of the analyte obtained by the Waters XBridge BEH Amide column was the best, and could effectively separate the four groups of isomers. L-alanine and sarcosine displayed the same protonated ion [M+H]^+^ at *m/z* 90.05502 and 90.05502, respectively, with the same molecular formula C_3_H_7_NO_2_. α-Aminobutyric acid, β-aminobutyric acid, and γ-aminobutyric acid displayed a similar protonated ion [M+H]^+^ at *m/z* 104.07050, 104.07061, and 104.07050, respectively, with the same molecular formula C_4_H_9_NO_2_. L-isoleucine and L-leucine displayed the same protonated ion [M+H]^+^ at *m/z* 132.10202 with the same molecular formula C_6_H_13_NO_2_. 1-Methyl-L-histidine and 3-methyl-L-histidine displayed a similar protonated ion [M+H]+ at *m/z* 170.09261 and 170.09272, respectively, with the same molecular formula C_7_H_11_N_3_O_2_. Therefore, the Waters XBridge BEH Amide column was selected as the analytical column.

This experiment also compared different mobile phase systems (water–acetonitrile, water–methanol, 0.1% formic acid water−0.1% formic acid acetonitrile, and 0.2% formic acid water−0.2% formic acid acetonitrile) to obtain better resolution and chromatographic separation ability. In the positive mode, the addition of formic acid provided more H^+^, which helped to enhance the response intensity of the target compound. When the organic phase of the mobile phase system was methanol and acetonitrile, the chromatographic response and peak shape of the target compound were not much different, but when methanol was used as the mobile phase, a longer equilibration time was required and the system pressure fluctuated greatly. Therefore, water containing 0.2% (v/v) formic acid and acetonitrile containing 0.2% (v/v) formic acid were selected as the mobile phase. Three different flow rates (0.3 mL/min, 0.4 mL/min, and 0.5 mL/min) were tested to obtain a fast and reliable separation. The results indicated that 0.4 mL/min was the optimal flow rate considering the resolution, run time, and column pressure. Under the optimal UPLC conditions, the extracted ion chromatograms of the 35 amino acids are presented in Figure 1.

### Method Validation

#### Linearity and Sensitivity

The calibration curves were generated from the relationship between the peak area and the concentration of target compounds with a concentration range of 0.5–1,000 μg/L. LOD was defined as the lowest concentration of free amino acids in sample, which can be detected but not quantified. LOQ was determined as the lowest concentration of free amino acids in sample, which can be quantified with acceptable precision and accuracy under the normal operating conditions of the method. A series of spiked samples was prepared to determine the LOD and LOQ when the signal-to-noise ratios of the 35 amino acids were about 3 and 10, respectively. As shown in Table 2, the linearity of the 35 amino acids were satisfactory with correlation coefficients (*R*^2^) higher than 0.9980. The LODs and LOQs of the 35 amino acids were 0.13–25.00 and 0.25–50.0 mg/kg, respectively.

**Table 2:** Linearity, LOD, and LOQ of 35 amino acids.

**Compound**	**Linear equation**	**Linear range** **(μg/L)**	***R* ^**2**^**	**LOD** **(mg/kg)**	**LOQ** **(mg/kg)**	Recovery (RSD)*		
						**50 mg/kg**	**150 mg/kg**	**500 mg/kg**
L-Arginine	Y = 712.49596X+1855.66828	2.5–1,000	0.99914	0.50	1.25	77.3 (4.73)	90.7 (3.66)	87.4 (3.90)
L-Aspartic acid	Y = 245.91896X+44.40999	20–1,000	0.99963	5.00	10.00	80.3 (9.10)	91.0 (7.52)	86.2 (5.53)
L-Cysteine	Y = 128.74993X-2483.03825	50–1,000	0.99944	12.5	25.00	80.0 (4.47)	89.4 (9.39)	96.3 (4.18)
L-Cystine	Y = 371.23366X+5246.96481	2.5–1,000	0.99954	0.50	1.25	83.3 (9.56)	91.8 (5.27)	95.8 (5.01)
L-Glutamic acid	Y = 392.30557X+10.05360	5–1,000	0.99962	1.25	2.50	72.7 (9.80)	92.9 (5.19)	99.7 (2.47)
Glycine	Y = 42.62232X+758.58867	100–1,000	0.99828	25.0	50.00	84.3 (3.49)	90.9 (6.06)	94.0 (4.99)
L-Histidine	Y = 652.69714X+2906.99013	2.5–1,000	0.99916	0.50	1.25	81.0 (4.62)	91.9 (8.96)	93.3 (4.58)
L-Isoleucine	Y = 400.87005X+7.90515e4	20–1,000	0.99834	5.00	10.00	76.3 (8.99)	90.4 (5.39)	89.6 (8.52)
L-Leucine	Y = 246.71219X+25428.48128	20–1,000	0.99895	5.00	10.00	80.3 (6.57)	88.6 (9.67)	89.7 (5.60)
L-Lysine	Y = 388.81504X-9916.88241	5–1,000	0.99944	1.25	2.50	88.0 (6.89)	89.2 (8.50)	91.9 (6.71)
L-Methionine	Y = 794.93701X-1225.64686	5–1,000	0.99951	1.25	2.50	82.3 (4.18)	95.0 (1.31)	100.8 (0.95)
L-Phenylalanine	Y=1047.32901X+3.00590e4	0.5-1000	0.99810	0.13	0.25	71.0 (10.2)	89.3 (7.17)	87.8 (5.38)
L-Proline	Y = 2785.34443X+15998.35998	2.5–1,000	0.99995	0.50	1.25	77.7 (8.53)	80.8 (8.97)	89.8 (5.25)
L-Serine	Y = 175.19613X-459.03363	10–1,000	0.99862	2.50	5.00	77.3 (8.60)	88.4 (8.94)	89.2 (7.78)
L-Threonine	Y = 319.78661X-640.30454	10–1,000	0.99941	2.50	5.00	77.0 (9.40)	82.3 (9.67)	88.1 (6.18)
L-Tyrosine	Y = 695.20787X+1382.31684	5–1,000	0.99988	1.25	2.50	80.3 (4.57)	105.1 (5.26)	95.3 (3.94)
L-Valine	Y = 660.48179X+19098.57317	15–1,000	0.99959	3.75	7.50	90.0 (7.57)	94.9 (2.50)	102.0 (1.23)
L-Alanine	Y = 111.59111X-1462.13625	20–1,000	0.99864	5.00	10.00	86.0 (4.16)	93.0 (2.21)	96.3 (4.37)
Theanine	Y = 1776.92273X+6744.45179	5–1,000	0.99984	1.25	2.50	79.7 (9.45)	94.6 (2.11)	92.1 (8.12)
L-Tryptophan	Y = 798.22194X+5509.98570	5–1,000	0.99989	1.25	2.50	89.0 (7.35)	92.7 (1.88)	101.8 (0.45)
L-Asparagine	Y = 271.81905X-678.93195	10–1,000	0.99944	2.50	5.00	79.7 (10.1)	90.0 (8.63)	92.6 (4.49)
L-Glutamine	Y = 268.81006X+3744.16195	5–1,000	0.99916	1.25	2.50	79.0 (11.0)	72.9 (7.47)	88.3 (3.11)
Aminoadipic acid	Y = 252.66609X-1009.17764	10–1,000	0.99864	2.50	5.00	84.7 (5.10)	91.4 (3.37)	91.6 (6.44)
α-Aminobutyric acid	Y = 30.95211X+2875.25568	50–1,000	0.99856	12.5	25.00	86.0 (6.06)	94.2 (4.28)	89.3 (0.89)
β-Aminobutyric acid	Y = 304.38085X-7311.81303	10–1,000	0.99897	2.50	5.00	86.7 (5.40)	92.4 (1.75)	70.1 (3.59)
γ-Aminobutyric acid	Y = 237.63091X	10–1,000	0.99920	2.50	5.00	73.7 (6.74)	89.9 (6.56)	89.1 (3.02)
Citrulline	Y = 326.91270X-359.40922	5–1,000	0.99919	1.25	2.50	96.3 (8.45)	93.7 (1.33)	93.6 (1.23)
Hydroxyproline	Y = 1188.51708X-10472.55309	5–1,000	0.99803	1.25	2.50	86.7 (4.30)	90.9 (7.36)	97.1 (2.20)
Hydroxylysine	Y = 147.61396X+158.38431	10–1,000	0.99949	2.50	5.00	88.3 (5.98)	97.0 (1.29)	95.1 (0.74)
1-Methyl-L-histidine	Y = 1959.69840X-8.65412e4	5–1,000	0.99817	1.25	2.50	88.0 (4.77)	97.9 (1.85)	95.9 (4.59)
3-Methyl-L-histidine	Y = 1096.39834X-18386.66480	5–1,000	0.99983	1.25	2.50	86.7 (9.53)	93.0 (5.24)	95.6 (1.88)
L-Ornithine	Y = 353.22736X-12881.75504	50–1,000	0.99975	12.5	25.00	82.7 (8.47)	90.2 (8.50)	88.5 (3.76)
Sarcosine	Y = 196.41758X+11684.55602	50–1,000	0.99953	12.5	25.00	86.7 (4.04)	85.9 (7.09)	85.0 (2.95)
DL-Homocysteine	Y = 142.21203X+6753.17341	50–1,000	0.99887	12.5	25.00	88.0 (5.75)	86.7 (6.88)	79.3 (8.59)
L-Pipecolic acid	Y = 4544.31048X-1525.44628	5–1,000	0.99857	1.25	2.50	73.0 (9.92)	89.2 (6.22)	92.0 (3.12)

* *Accuracy expressed as average trueness of the calculated (quantified) concentration value, and relative standard deviation (RSD) of n = 6 replicate samples*.

#### Recovery and Precision

The recovery and precision experiments were carried out by adding 35 amino acid standard solutions to the known amino acid content of tea sample. Samples spiked at three different concentrations (50, 150, and 500 mg/kg) for each of the amino acids, and each concentration was repeated six times. As shown in Table 2, the average recovery rate ranged from 70.1 to 105.1%, and the RSDs ranged from 0.45 to 11.0%. The recoveries were considered acceptable data to the method, and the results indicated that the precision was reasonable.

### Application to Actual Samples

In order to investigate the content of the 35 amino acids in tea, nine tea samples from local tea shops were analyzed using the established method in this research, and each sample was repeated three times. The results (Table 3) showed that the compositions and contents of 35 amino acids were different in the nine tea samples. Except for L-cysteine, L-cystine, glycine, aminoadipic acid, α-aminobutyric acid, β-aminobutyric acid, hydroxylysine, L-ornithine, and DL-homocysteine, the content of amino acids were higher than LOQs. There was little difference in the types of amino acids in different teas. Theanine was the dominating amino acid in tea infusions of different cultivars, followed by L-arginine, L-aspartic acid, L-glutamine, L-valine, L-glutamic acid, and L-asparagine. L-Phenylalanine is associated with the intensity of bitter and astringent taste of the tea. The content of L-phenylalanine in green tea was higher than in black tea. It showed that the quantity of phenylalanine could lay a foundation to assess the bitter and astringent taste of the tea. For green tea, theanine content varied from 3.844 to 11.638 mg/g, which is consistent with the literature results (37, 38). The theanine content in green tea was higher than that in black tea (2.787 mg/g). Furthermore, a clear corresponding relationship between the content of amino acid and the elaboration process of tea could be observed. The total amount of amino acids in black tea was lower than that in green tea, which may be due to the degradation of amino acids during the fermentation process. Green tea (non-fermented) and black tea (fully fermented) are the two major commercial types of tea. Green tea is derived directly from inactivating by steaming or microwave and drying the fresh tea leaves. For black tea, most of tea catechins are enzymatic oxidized and polymerized during the fermentation process. Accompanying the fermentation process, the free amino acids in tea leaves may take great changes. Accordingly, black teas contain less free amino acids compared with green tea (39).

**Table 3:** Contents^*^ of free amino acids in different types of tea.

**Compound**	**Congou black tea**	**Yellow mountain fuzz tip**	**Rizhao green tea**	**Green tea 1**	**Green tea 2**	**Maojian tea**	**Yacca tea**	**Taiping houkui tea**	**Sword shaped green tea**
L-Arginine	0.203 ± 0.002	1.213 ± 0.039	0.482 ± 0.009	1.339 ± 0.034	1.466 ± 0.035	0.704 ± 0.020	0.875 ± 0.028	0.908 ± 0.009	1.091 ± 0.010
L-Aspartic acid	0.812 ± 0.018	2.373 ± 0.001	2.380 ± 0.012	2.302 ± 0.026	2.565 ± 0.009	2.435 ± 0.005	1.951 ± 0.025	2.218 ± 0.001	3.343 ± 0.037
L-Cysteine	ND	ND	ND	ND	ND	ND	ND	ND	ND
L-Cystine	ND	ND	ND	ND	ND	ND	ND	ND	ND
L-Glutamic acid	0.507 ± 0.003	2.592 ± 0.002	1.712 ± 0.016	2.953 ± 0.000	2.492 ± 0.033	2.003 ± 0.005	4.170 ± 0.018	3.024 ± 0.014	2.948 ± 0.025
Glycine	ND	ND	ND	ND	ND	ND	ND	ND	ND
L-Histidine	0.036 ± 0.002	0.149 ± 0.006	0.146 ± 0.003	0.092 ± 0.003	0.209 ± 0.001	0.143 ± 0.000	0.449 ± 0.010	0.120 ± 0.004	0.376 ± 0.006
L-Isoleucine	0.180 ± 0.011	0.284 ± 0.008	0.369 ± 0.007	0.326 ± 0.009	0.577 ± 0.007	0.427 ± 0.004	0.770 ± 0.002	0.217 ± 0.004	0.735 ± 0.006
L-Leucine	0.232 ± 0.011	0.363 ± 0.007	0.343 ± 0.014	0.324 ± 0.007	0.486 ± 0.006	0.333 ± 0.005	0.581 ± 0.005	0.323 ± 0.006	0.649 ± 0.003
L-Lysine	0.126 ± 0.001	0.240 ± 0.002	0.334 ± 0.006	0.341 ± 0.009	0.528 ± 0.001	0.361 ± 0.004	0.786 ± 0.009	0.228 ± 0.006	0.766 ± 0.013
L-Methionine	0.038 ± 0.005	0.036 ± 0.001	0.022 ± 0.001	0.022 ± 0.000	0.031 ± 0.001	0.021 ± 0.001	0.039 ± 0.001	0.037 ± 0.001	0.033 ± 0.000
L-Phenylalanine	0.290 ± 0.002	0.523 ± 0.005	0.557 ± 0.116	0.336 ± 0.015	0.410 ± 0.010	0.640 ± 0.006	0.610 ± 0.024	0.379 ± 0.009	0.437 ± 0.003
L-Proline	0.051 ± 0.000	0.029 ± 0.001	0.085 ± 0.001	0.059 ± 0.001	0.114 ± 0.002	0.079 ± 0.001	0.173 ± 0.000	0.037 ± 0.001	0.183 ± 0.002
L-Serine	0.305 ± 0.013	0.332 ± 0.007	0.539 ± 0.006	0.578 ± 0.008	0.684 ± 0.007	0.486 ± 0.004	0.746 ± 0.011	0.411 ± 0.004	0.784 ± 0.008
L-Threonine	0.164 ± 0.004	0.566 ± 0.002	0.610 ± 0.002	0.508 ± 0.000	0.740 ± 0.012	0.578 ± 0.004	0.965 ± 0.002	0.536 ± 0.001	0.951 ± 0.007
L-Tyrosine	0.051 ± 0.003	0.145 ± 0.002	0.074 ± 0.031	0.133 ± 0.003	0.185 ± 0.003	0.151 ± 0.003	0.279 ± 0.003	0.127 ± 0.001	0.165 ± 0.058
L-Valine	3.495 ± 0.013	3.182 ± 0.047	5.153 ± 0.013	3.235 ± 0.035	3.216 ± 0.001	5.009 ± 0.031	5.848 ± 0.038	2.233 ± 0.042	7.403 ± 0.078
L-Alanine	0.217 ± 0.005	0.150 ± 0.000	0.111 ± 0.001	0.190 ± 0.003	0.194 ± 0.002	0.183 ± 0.003	0.212 ± 0.007	0.163 ± 0.010	0.181 ± 0.005
Theanine	2.787 ± 0.009	11.638 ± 0.198	5.330 ± 0.020	5.775 ± 0.170	5.843 ± 0.023	4.499 ± 0.024	5.745 ± 0.040	9.073 ± 0.008	3.844 ± 0.010
L-Tryptophan	0.102 ± 0.001	0.258 ± 0.005	0.188 ± 0.001	0.160 ± 0.001	0.131 ± 0.042	0.236 ± 0.003	0.202 ± 0.006	0.206 ± 0.009	0.195 ± 0.001
L-Asparagine	0.254 ± 0.001	0.629 ± 0.001	1.354 ± 0.004	0.607 ± 0.005	1.303 ± 0.001	1.154 ± 0.028	3.248 ± 0.025	0.401 ± 0.007	1.987 ± 0.001
L-Glutamine	0.311 ± 0.002	5.773 ± 0.098	0.793 ± 0.014	1.637 ± 0.003	2.647 ± 0.005	1.949 ± 0.002	2.265 ± 0.040	5.168 ± 0.028	1.931 ± 0.005
Aminoadipic acid	ND	ND	ND	ND	ND	ND	ND	ND	ND
α-Aminobutyric acid	ND	ND	ND	ND	ND	ND	ND	ND	ND
β-Aminobutyric acid	ND	ND	ND	ND	ND	ND	ND	ND	ND
γ-Aminobutyric acid	0.059 ± 0.005	0.119 ± 0.004	0.017 ± 0.002	0.072 ± 0.003	0.073 ± 0.005	0.052 ± 0.003	0.051 ± 0.004	0.052 ± 0.005	0.045 ± 0.003
Citrulline	0.004 ± 0.000	0.029 ± 0.000	0.015 ± 0.000	0.023 ± 0.001	0.041 ± 0.000	0.011 ± 0.001	0.012 ± 0.000	0.026 ± 0.001	0.036 ± 0.001
Hydroxyproline	0.017 ± 0.000	0.020 ± 0.000	0.024 ± 0.000	0.029 ± 0.000	0.020 ± 0.000	0.021 ± 0.000	0.019 ± 0.000	0.022 ± 0.000	0.020 ± 0.000
Hydroxylysine	ND	ND	ND	ND	ND	ND	ND	ND	ND
1-Methyl-L-histidine	0.013 ± 0.000	0.015 ± 0.000	0.016 ± 0.000	0.014 ± 0.000	0.017 ± 0.000	0.015 ± 0.000	0.018 ± 0.000	0.016 ± 0.000	0.021 ± 0.000
3-Methyl-L-histidine	ND	0.009 ± 0.000	0.011 ± 0.000	0.010 ± 0.001	0.012 ± 0.000	0.009 ± 0.000	0.016 ± 0.000	0.010 ± 0.001	0.031 ± 0.001
L-Ornithine	ND	ND	ND	ND	ND	ND	ND	ND	ND
Sarcosine	ND	0.018 ± 0.000	ND	ND	ND	ND	ND	ND	ND
DL-Homocysteine	ND	ND	ND	ND	ND	ND	ND	ND	ND
L-Pipecolic acid	0.045 ± 0.001	0.083 ± 0.001	0.117 ± 0.003	0.117 ± 0.001	0.182 ± 0.002	0.133 ± 0.003	0.281 ± 0.001	0.081 ± 0.003	0.269 ± 0.007
Total	10.299 ± 0.041	30.765 ± 0.371	20.782 ± 0.106	21.181 ± 0.307	24.163 ± 0.160	21.631 ± 0.092	30.311 ± 0.119	26.018 ± 0.105	28.422 ± 0.187

* *Value (mg/g) = mean ± SD (n = 3). ND, not detectable*.

Compared to other available methods for the same analytical determination (37, 40–44), this method measured the largest number of amino acids. At the same time, the method did not need derivatization, the pretreatment method was simple, the analysis time was short, and the selectivity was high.

## Conclusion

An accurate, simple, and sensitive method for direct determination of 35 free amino acids in tea based on UPLC-Q-TOF/MS has been established. One injection can achieve both TOF–MS and IDA–MS/MS detection, and obtain the accurate mass, isotope distribution, and secondary ion fragment information. The UPLC-Q-TOF/MS is not affected by matrix effect, and the qualitative results can be confirmed by using the secondary ion fragmentation spectrum. It can be widely used for the rapid determination of free amino acids in tea, and provides a reference for the determination of amino acids in other plant tissues. This method also provides technical support for tea quality analysis and research.

## Data Availability Statement

The original contributions presented in the study are included in the article/Supplementary Material, further inquiries can be directed to the corresponding author/s.

## Author Contributions

YZ and LZ conceived and designed the experiments. JL, JM, QL, and SF performed the experiments. LF and HM analyzed the data. JL and JM wrote the original draft. All authors have read and approved the manuscript.

## Funding

This work was supported by the National Key Research and Development Plan of China (2016YFD0401104).

## Conflict of Interest

The authors declare that the research was conducted in the absence of any commercial or financial relationships that could be construed as a potential conflict of interest.

## Publisher's Note

All claims expressed in this article are solely those of the authors and do not necessarily represent those of their affiliated organizations, or those of the publisher, the editors and the reviewers. Any product that may be evaluated in this article, or claim that may be made by its manufacturer, is not guaranteed or endorsed by the publisher.
