# Supplementary material for: Determination of 35 Free Amino Acids in Tea Using Ultra-Performance Liquid Chromatography Coupled With Quadrupole Time-of-Flight Mass Spectrometry
Source: Front Nutr. 2021 Dec 1;8:767801. doi: 10.3389/fnut.2021.767801 (PMC8697017; doi:10.3389/fnut.2021.767801)
Supplement: Supplementary file 1 [file Data_Sheet_1.docx]

Determination of 35 free amino acids in tea using UPLC-Q-TOF/MS

**Jian Li^1,3†^, Junmei Ma^2,4†^, Qiang Li^2^, Sufang Fan^2^, Lixin Fan^2^, Hongyu Ma^2^, Yan Zhang^2,4*^ and Lei Zheng^1^***

^1^School of Food and Biological Engineering, Hefei University of Technology, Hefei, China.

^2^Hebei Food Safety Key Laboratory, Hebei Food Inspection and Research Institute, Shijiazhuang, China.

^3^College of Applied Arts and Science, Beijing Union University, Beijing, China.

^4^Hebei Key Laboratory of Forensic Medicine, College of Forensic Medicine, Hebei Medical University, Shijiazhuang, China.

*** Correspondence:**Corresponding Author
snowwinglv@126.com, [lei.zheng@aliyun.com](mailto:lei.zheng@aliyun.com)

†These authors have contributed equally to this work and share first authorship

|   L-Arginine |
| --- |
|   L-Aspartic acid |
|   L-Cysteine |
|   L-Cystine |
|   L-Glutamic acid |
|   Glycine |
|   L-Histidine |
|   L-Isoleucine |
|   L-Leucine |
|   L-Methionine  L-Lysine |
|  |
|   L-Phenylalanine |
|   L-Proline |
|   L-Threonine  L-Serine |
|   L-Tyrosine |
|  |
|   L-Valine |
|   Sarcosine |
|   L-Alanine |
|   Theanine |
|   L-Tryptophan |
|   L-Asparagine |
|   Aminoadipic acid  L-Glutamine |
|  |
|   β-Aminobutyric acid |
|   γ-Aminobutyric acid |
|   α-Aminobutyric acid |
|   Citrulline |
|   Hydroxyproline |
|   Hydroxylysine |
|   1-Methyl-L-histidine |
|   3-Methyl-L-histidine |
|   L-Ornithine |
|   DL-Homocysteine |
|   L-Pipecolic acid |

Figure S1 MS/MS spectra of the 35 amino acids.

Figure S2 Extracted ion chromatograms of the 35 amino acids precursor ions under different chromatographic columns. (A) Waters Acquity UPLC HSS T3 (2.1 mm×100 mm, 1.8 μm) chromatographic columns. (B) Waters XBridge BEH C_18_ (2.1 mm×100 mm, 2.5 μm) chromatographic columns. (C) Waters Cortecs UPLC HILIC (2.1 mm×100 mm, 1.6 μm)chromatographic columns.

.
